# Highly Concentrated, Conductive, Defect-free Graphene Ink for Screen-Printed Sensor Application

**DOI:** 10.1007/s40820-021-00617-3

**Published:** 2021-03-08

**Authors:** Dong Seok Kim, Jae-Min Jeong, Hong Jun Park, Yeong Kyun Kim, Kyoung G. Lee, Bong Gill Choi

**Affiliations:** 1 Department of Chemical Engineering, Kangwon National University, Samcheok, Gangwon-do 25913 Republic of Korea; 2 Resources Utilization Research Center, Korea Institute of Geoscience and Mineral Resources, Daejeon, 34132 Republic of Korea; 3 Center for Nano Bio Development, National Nanofab Center, Daejeon, 34141 Republic of Korea

**Keywords:** Graphene ink, Fluid dynamics, Screen printing, Ion sensor, Real-time monitoring

## Abstract

**Highlights:**

- Ultrathin and defect-free graphene ink is prepared through a high-throughput fluid dynamics process, resulting in a high exfoliation yield (53.5%) and a high concentration (47.5 mg mL^−1^).
- A screen-printed graphene conductor exhibits a high electrical conductivity of 1.49 × 10^4^ S m^−1^ and good mechanical flexibility.
- An electrochemical sodium ion sensor based on graphene ink exhibits an excellent potentiometric sensing performance in a mechanically bent state.
- Real-time monitoring of sodium ion concentration in sweat is demonstrated.

**Abstract:**

Conductive inks based on graphene materials have received significant attention for the fabrication of a wide range of printed and flexible devices. However, the application of graphene fillers is limited by their restricted mass production and the low concentration of their suspensions. In this study, a highly concentrated and conductive ink based on defect-free graphene was developed by a scalable fluid dynamics process. A high shear exfoliation and mixing process enabled the production of graphene at a high concentration of 47.5 mg mL^−1^ for graphene ink. The screen-printed graphene conductor exhibits a high electrical conductivity of 1.49 × 10^4^ S m^−1^ and maintains high conductivity under mechanical bending, compressing, and fatigue tests. Based on the as-prepared graphene ink, a printed electrochemical sodium ion (Na^+^) sensor that shows high potentiometric sensing performance was fabricated. Further, by integrating a wireless electronic module, a prototype Na^+^-sensing watch is demonstrated for the real-time monitoring of the sodium ion concentration in human sweat during the indoor exercise of a volunteer. The scalable and efficient procedure for the preparation of graphene ink presented in this work is very promising for the low-cost, reproducible, and large-scale printing of flexible and wearable electronic devices. 
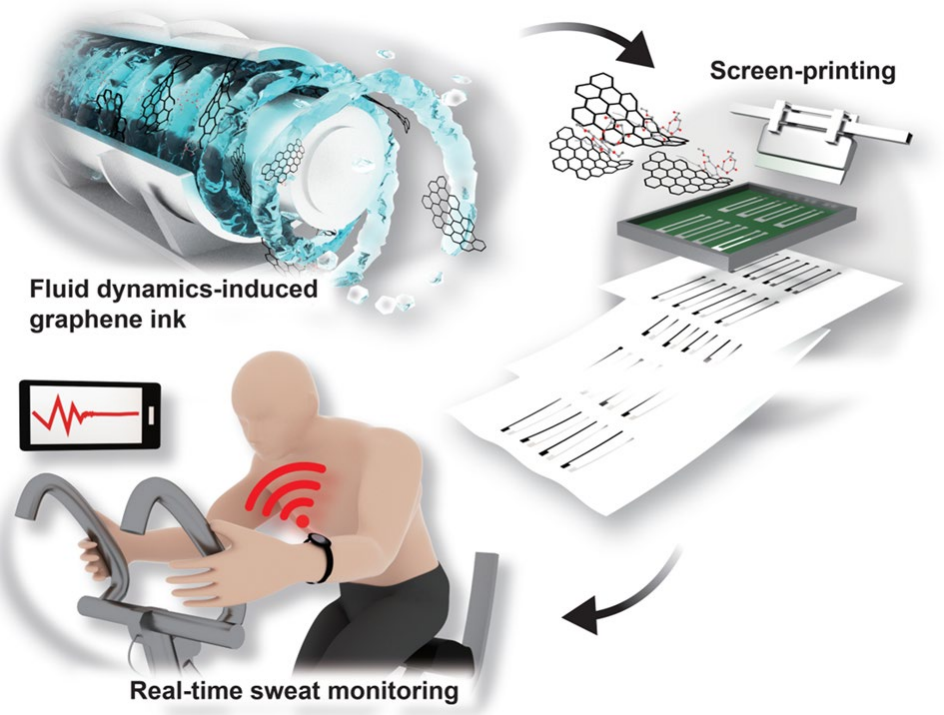

**Supplementary Information:**

The online version contains supplementary material available at 10.1007/s40820-021-00617-3.

## Introduction

Wearable chemical sensor technology has rapidly developed in recent years with applications in personalized healthcare, fitness management, and medical diagnostics because it provides continuous and real-time physiological information with regard to health status changes to the wearer [1–5]. A wearable wireless sensor device typically contains the following key functional units: a substrate, active materials, and an integrated circuit module [6, 7]. The active materials, composed of sensing and electrical components, are printed or transferred to flexible or stretchable substrates such as flexible plastics, paper, textiles, and soft polymer films [8, 9]. To further provide flexibility or stretchability to sensor devices, mechanically structured design approaches have been used in the manufacture of wearable devices, including wavy, fractal, origami, kirigami, and island-interconnected structures [10–14]. The as-obtained sensing platforms selectively recognize human body fluids such as saliva, tear, sweat, and urine as biochemical markers, and the collected data can be transmitted to the user’s smartphone application [15, 16]. However, saliva, tear, and urine-based sensors are difficult for continuous and noninvasive health monitoring in a wearable platform because of limited location and sample conditions [17–20]. In contrast, sweat is easy to access noninvasively through the skin and is rich in important analytes, such as electrolytes (e.g., Na^+^, Cl^−^, K^+^, and pH) and metabolites (e.g., glucose, lactate, and cortisol) [21, 22]. Recent studies have shown that potentiometric ion sensors in a wearable format are useful for real-time monitoring of dynamic changes in ion concentrations in sweat during an individual’s activities [23, 24]. The on-body monitoring of sodium and chloride ions in sweat can be utilized to indicate dehydration status and to diagnose cystic fibrosis [25, 26]. The potassium level in sweat is related to muscle activity [27], and pH monitoring can be used for the management of chronic wounds [28]. A number of wearable sweat sensors have recently been developed in many different device forms, such as multi-sensor arrays [29], textile-based potentiometric sensors [30], smart bandages [31], patches [32], and tattoos [33], and these sensors are capable of monitoring electrolytes, metabolites, heavy metals, and toxic gases in human body fluids [34–36]. Despite the development of state-of-the-art sensors, considering their many applications in point-of-care diagnostics and exercise monitoring, the wearable sensor market requires that the sensor platforms are low cost, disposable, and amenable to mass production with high reproducibility.

Screen printing is a high-speed, cost-effective, reproducible, and large-scale process for the fabrication of sensor electrodes or devices by the transfer of inks onto flexible substrates through a stencil mask [37, 38]. The properties of functional inks, such as their conductivity, concentration, viscosity, and flexibility, are key factors for realizing high-performance wearable chemical sensors [39, 40]. Graphene, a two-dimensional carbon nanosheet, has gained considerable attention as a promising ink material for the construction of next-generation printed flexible electronics and devices because of its outstanding mechanical, thermal, chemical, and electrical properties [40–42]. Graphene materials are typically prepared from graphite by chemical oxidative exfoliation and liquid-phase exfoliation (LPE) methods [43, 44]. The oxidation of graphite and subsequent exfoliation results in a large quantity of graphene oxide (GO) nanosheets dispersed in polar solvents such as water and alcohols [43]. An insulating GO can be converted into conductive reduced graphene oxide (RGO) by chemical or annealing reduction [45]. However, the unrepaired defects and distorted structure of RGO lead to low electrical conductivity, thus limiting its application in conductive inks. In this regard, there has been much research focused on the direct delamination of graphite by the LPE method to potentially obtain suspensions of defect-free graphene without any chemical functionalization [46–48]. The resultant graphene is used in the form of a highly conductive ink, paste, or composite for printing many devices [49, 50]. However, LPE still provides a low yield (< 12%) or graphene concentration (< 10 mg mL^−1^), which is a significant limitation in the preparation of exfoliated graphene [51, 52]. Although a low-power ultrasonication resulted in a high yield of 33% for preparation of defect-free single-layer graphene for inkjet-printed graphene electronics, a long reaction time of 9 h and a low concentration of ~ 0.11 g L^−1^ limit this to large-scale screen-printing applications [53]. The use of an ultrasonic bath or probe in LPE is time-consuming because of its energy is applied locally and non-homogeneously [54, 55], and thus, the exfoliation process is not suitable for industrial scale-up. Moreover, the concentration of graphene needs to be increased in order to develop screen-printing inks through post-processing procedures such as repetitive centrifugation and redispersion, which increase the preparation time. To address these issues, the fluid dynamics process has emerged as a rapid and scalable method to produce defect-free and large-lateral size graphene sheets from direct exfoliation of graphite using suitable solvents and water [51, 56]. Typically, graphite flakes are delaminated by fluid dynamics-induced shear exfoliation. Other factors such as pressure, cavitation, and collision and efficient mixing behavior contribute to the exfoliation process. To date, various fluid dynamics-based exfoliation approaches have been attempted for graphite exfoliation, including vortex fluidic flow, pressure-driven fluid dynamics, Taylor–Couette (TC) flow, and mixer-driven fluid dynamics. In particular, a TC flow generated in the small gap between two rotating cylinders provides high wall shear stress and pressure and efficient mass transfer, and thus, these fluidic features can efficiently produce defect-free and few-layer graphene sheets in a large quantity.

Here, we report the scalable and efficient production of highly concentrated and conductive inks of defect-free graphene by a TC flow-induced exfoliation and mixing process. The delamination of graphite resulted in a high yield of exfoliated graphene (ex-Gr) of 53.5%, which was obtained at a high suspension concentration of 47.5 mg mL^−1^. The screen-printed ex-Gr conductor exhibited a high electrical conductivity of 1.49 × 10^4^ S m^−1^ and good mechanical flexibility. Moreover, we demonstrated that the printed ex-Gr conductor can be used as an electrochemical ion-sensing electrode for the potentiometric detection of sodium ions (Na^+^). The resulting Na^+^ sensor exhibited high sensitivity, fast response time, good repeatability, and good selectivity. As a proof-of-concept, we performed on-body testing on a volunteer wearing the wireless electronically integrated Na^+^-sensing device during a stationary bike exercise to demonstrate the real-time monitoring of Na^+^ concentration in his sweat.

## Experimental

### Materials

Sodium ionophore X, sodium tetrakis[3,5-bis(trifluoromethyl)phenyl]borate (Na-BARF), bis(2-ethylhexyl) sebacate (DOS), poly(vinyl chloride) (PVC) with high molecular weight, sodium chloride, potassium chloride, magnesium chloride, calcium chloride, ammonium chloride, ethyl cellulose with viscosity 10 cP, terpineol, BUTVAR® B-98 (polyvinyl butyral, PVB), methanol, and tetrahydrofuran (THF) were purchased from Sigma Aldrich. Ethyl alcohol was purchased from Samchun Chemicals Co. Ltd. Ag and Ag/AgCl inks were obtained from Asahi chemical research laboratory Co. Ltd. Deionized (DI) water was prepared by using a Milli-Q unit (resistivity: 18.2 MΩ cm^−1^, Millipore System, Darmstadt, Germany).

### Preparation of ex-Gr Inks

The conductive and defect-free ex-Gr inks were prepared by the fluid dynamics-assisted exfoliation and mixing process. A mixture of graphite and EC (9:1 in weight) dispersed in terpineol/ethanol (5:5 in volume) was loaded in a reactor and processed at 2000 rpm for 2 h. The fluidic reactor is made by combining two inner-rotational and outer-stationary cylinders based on stainless steel. At a specific rotation speed of 2000 rpm, the reactor generates the Taylor vortexes entire reactor volume that provides unique mass transfer and shear field [57]. Different concentrations of graphite (1, 5, 10, 50, 100, and 200 mg mL^−1^) were tested. After the exfoliating and mixing process, the exfoliated dispersion was collected by centrifugation at 250 g for 30 min. And then, the ultra-small size of graphene was also removed by centrifugation at 3200 g for 60 min. The as-obtained ex-Gr inks were heated at 225 °C for 24 h under vacuum to remove terpineol and ethyl cellulose, and then weighed by using an electronic scale XSR105 (Mettler toledo) to evaluate the exfoliation yield and final concentration of ex-Gr nanosheets.

### Preparation of Na^+^ Sensor Based on Screen-Printed ex-Gr Electrode

Prior to fabricating the Na^+^ sensors, the electrical circuit electrodes were prepared using a screen printer SJ-7450S (Sung-Jin tech Ltd., Korea) and a custom stainless-steel mask designed with two-electrode patterns. The ex-Gr and Ag inks were printed onto a flexible substrate of polyethylene terephthalate (PET) film, in which ex-Gr and Ag inks were used as electrical circuits for the working and reference electrodes, respectively. The resultant electrode was annealed at 200 °C for 120 min. Custom stainless-steel masks were made with different thicknesses of 25‒45 µm, corresponding to the thickness of the printed ex-Gr conductors in 20‒41 µm. The working electrodes were prepared by coating the Na^+^-ISM onto the surface of printed ex-Gr. The Na^+^-ISM is consisted of Na ionophore X (1% weight by weight, w/w), Na-BARF (0.5%, w/w), PVC (31.5%, w/w), and DOS (67%, w/w) in THF (1 mL). For a reference electrode, Ag/AgCl ink was coated onto the reaction part of the printed Ag-electrical circuit. To ensure electrochemical stability of the reference electrode, the as-obtained Ag/AgCl-coated electrode was coated by a casting method using a mixture 50 mg of NaCl and 78 mg of PVB dissolved in 1 mL methanol solution [5, 58]. The resultant Na^+^ sensors were dried at room temperature for 24 h.

### Characterization and Measurements

Field-emission scanning electron microscope (FE-SEM, Hitachi S-4800) and field-emission transmission electron microscope (FE-TEM, JEOL Ltd. JEM-2100F HR) were performed to investigate the morphology and microstructure of ex-Gr nanosheets and printed ex-Gr electrodes. The atomic force microscopy (AFM) images were obtained by an AFM-Raman spectrometer (INNOVA-LABRAM HR800, Bruker Co., Ltd.). High-resolution X-ray diffraction (XRD) was performed on a D/MAX-2500 V with a *θ*/*θ* goniometer equipped with a Cu Kα radiation generator. X-ray photoelectron spectroscopy (XPS, Thermo MultiLab 20,000) and Raman microscopy (ARAMIS, Horiba Jobin Yvon) were performed to investigate chemical structure of graphite and ex-Gr. The electrical conductivity of printed ex-Gr conductors was measured using four-point probe system (Conductivity meter, Loresta-GX MCP-T700). The ex-Gr inks were screen-printed onto various substrates of PET, polyimide (PI), and paper, followed by annealing to different temperature (100, 125, 200, and 225 °C). For measure electrical conductivity, the as-obtained ex-Gr conductors were cut to 1 cm × 1 cm size. The electrochemical performances of Na^+^ sensors were evaluated using an electrochemical instrument of CHI 760 E (CH Instruments, USA). The different concentrations of NaCl solution (10^−1^‒10^−4^ M) were prepared by diluting 1 M NaCl solution. Fatigue test and bending test were performed using digital multimeters (Protek HC-81). Bending and compressing tests were measured resistance of bending angles while reduced length using ex-Gr screen-printed electrodes of 10 × 1 cm^2^ size. Fatigue test was measured resistance while bending over 1400 cycles. Cyclic voltammetric curves of screen-printed graphite and ex-Gr electrodes were measured at a scan rate of 50 mV s^−1^ in 10^−1^ M NaCl. Sensitivity of bending and normal state was measured different concentration of NaCl solutions (10^−1^‒10^−4^ M). Long-term stability of sodium sensor was measured for 15 h in 10^−1^ M NaCl solution. Ion selectivity of sodium sensor was evaluated using various ion solutions that is 10‒20 mM NaCl, 5 mM KCl, 0.5 mM NH_4_Cl, 0.5 mM MgCl_2_, and 0.5 mM CaCl_2_. The repeatability test of the Na^+^ sensor was performed using a titrated cycle of 10^−1^ to 10^−2^ to 10^−3^ to 10^−4^ M, in which the sensors were used continuously without washing steps.

### On-body Sweat Measurements

A wristwatch-type wearable device was fabricated by integrating Na^+^ sensor and a wireless electronic printed circuit board (PCB) module into 3D printed wristwatch. The PCB system was fabricated according to our previous reports [58, 59]. A block diagram of PCB system is described in Fig. S1. The PCB consists of the electrical units and communication modules, involving electrochemical ion sensor, interface circuits, a microcontroller unit (64 MHz, Arm Cortex-M4), a regulator (LP5907-3.3), an analog to digital converter (ADC, ADS 1114), and Bluetooth low energy (BLE). A lithium-ion battery (3.7 V) was used as the power source. The Na^+^ sensor was connected with PCB module using an electrical jumper wire (male to female). The Kapton tape for the insulation of Na^+^ sensor was attached at both end sides of the electrodes and PCB module. The PCB collects continuously the Na^+^ concentration data in sweat and transfer to a smartphone via Bluetooth. The real-time monitored Na^+^ concentration was displayed in a mobile application. On-body tests were according to compliance with relevant guidelines approved by the institutional review board of Kangwon National University (KWNUIRB-2018-12-002-001). A healthy volunteer (male aged 28-years-old) provided written informed consent after understanding the on-body test guidelines. A commercial heart rate sensor device was used together. Prior to on-body sweat test, a volunteer’s wrist was cleaned with soap and water and then dried using alcohol. Real-time monitoring of sweat sensors was performed by exercising on a stationary bike for 20 min at room temperature.

## Results and Discussion

### Preparation and Characterization of ex-Gr Inks

**Fig. 1:**
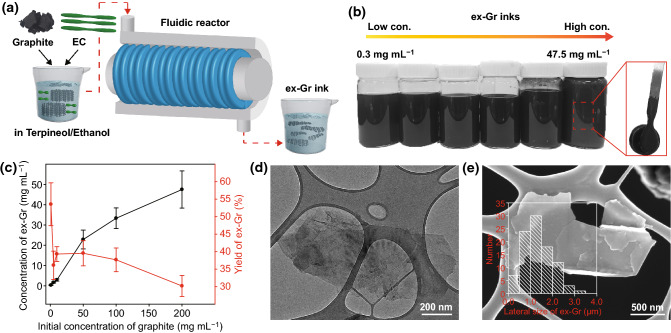
**a** Schematic illustration of the fluid dynamics process used for the preparation of ex-Gr ink. **b** Photograph image of the ex-Gr inks at different concentrations (0.3‒47.5 mg mL^−1^). **c** Concentrations and yields of the ex-Gr obtained at different initial concentrations of the graphite (1‒200 mg mL^−1^). **d** TEM and **e** SEM images of the ex-Gr sheet (Inset: histogram for the lateral size distribution of ex-Gr)

Figure 1a shows the simple and efficient preparation process for conductive ex-Gr inks. As an exfoliation method, a fluid dynamics-induced delamination process was employed to obtain a high yield of defect-free ex-Gr at a high concentration in the suspension. The use of the TC flow allowed uniform and high shear rates and fast mass transfer, resulting in high-throughput production of ex-Gr dispersed in suitable solvents [60]. Typically, a mixture of graphite and EC (9:1 in weight) dispersed in terpineol/ethanol (5:5 in volume) was loaded in a fluid dynamic reactor and processed for 2 h. The process was investigated with different concentrations of graphite (1, 5, 10, 50, 100, and 200 mg mL^−1^). EC was used as a stabilizer to efficiently exfoliate and suspend graphene flakes in an organic solvent such as a mixture of terpineol and ethanol. EC also served as a binder; it enhanced adhesion between graphene and printing substrates. After centrifugation to remove unexfoliated graphite, a large quantity of high-quality ex-Gr was obtained. The diverse fluid dynamics forces, including shear, pressure, cavitation, and collision forces [57], enable the efficient and rapid exfoliation of graphite, resulting in a high yield and high concentration of ex-Gr suspension. The yield and concentration of ex-Gr depended on the initial graphite concentration. The maximum yield of the as-obtained ex-Gr suspension of 53.5% was obtained at a low initial graphite concentration of 1 mg mL^−1^, and the concentration reached as high as 47.5 mg mL^−1^ when the initial graphite concentration was 200 mg mL^−1^ (Fig. 1b, c). A highly viscous and concentrated graphite loading over 200 mg mL^−1^ did not allow us to provide a TC-induced exfoliation process because a high viscous flow limits the rotating cylinders to a small distance between the two cylinders. Remarkably, the use of a fluid dynamics reactor enabled us to obtain highly conductive and concentrated graphene suspension at a large-scale and at a fast speed. A high exfoliation yield of 53.5% and a high concentration of 47.5 mg mL^−1^ of this process are notably better than that of conventional methods such as sonication, ball-milling, shearing, and processing with a mortar (Table S1, Supporting Information) [51, 60]. TEM images reveal highly exfoliated nanosheets and high-quality crystalline structure of ex-Gr (Fig. 1d). The flake size of ex-Gr was statistically analyzed from SEM images (Fig. 1e). For SEM observation, the ex-Gr suspension was drop-casted onto a TEM grid, and was then washed and annealed to remove the solvents and surfactant. According to the analysis of more than 90 sheets in the SEM images, the lateral size distribution of ex-Gr is in the range 0.29‒3.25 µm and its average lateral size is 1.10 ± 0.84 µm. The thickness of ex-Gr was determined to be 1.2 ± 0.8 nm via AFM measurement (Fig. S2). Based on this result, the as-obtained ex-Gr consists of few-layer nanosheets less than five layers, indicating multilayered graphene. The lateral size and thickness of few-layered ex-Gr obtained from the TC flow-induced exfoliation process were similar to previously reported values obtained from shearing and microfluidic exfoliation processes [61–65].

**Fig. 2:**
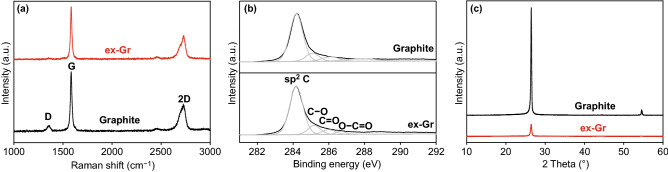
**a** Raman spectra, **b** XPS C 1 s spectra, and **c** XRD patterns of graphite and ex-Gr

The crystallinity and chemical quality of ex-Gr was investigated by Raman spectroscopy, XPS, and XRD. The Raman spectrum of ex-Gr has three characteristic peaks at 1357, 1582, and 2725 cm^‒1^, corresponding to the D, G, and 2D bands (Fig. 2a) [66]. The analysis of the Raman D/G band intensity ratio *(I*_D_/*I*_G_) allows us to obtain information on the extent of structural disorder or defects in ex-Gr sheets [67]. The value of *I*_D_/*I*_G_ was estimated to be 0.21, which is much lower than the previously reported values of 0.3‒2.5 for graphene materials obtained by the sonication-based LPE methods [54]. This result indicates the absence of basal-plane defects in the ex-Gr sheets. Compared to the 2D peak of graphite, ex-Gr exhibited a more symmetrical and broader 2D peak; that is, the full width at half maximum increased from 98 cm^−1^ (graphite) to 115.5 cm^−1^ (ex-Gr). This result indicates that the ex-Gr sheets predominantly have a mono- or few-layer structure [68]. The high-resolution C 1s XPS profile (Fig. 2b) of ex-Gr shows a sharp and prominent peak at 284.18 eV corresponding to the C‒C bond of graphite [67]. After deconvolution, the C 1s peak showed no peaks of oxygenated carbon (e.g., C‒O and C=O) [67]. Compared to XRD pattern of graphite, ex-Gr exhibited significantly decreased diffraction peak, indicating the presence of abundant exfoliated graphene sheets (Fig. 2c) [60]. According to these results, the fluid dynamics process yields ultrathin exfoliated graphene sheets with a high-quality graphitic structure.

### Electrical and Mechanical Characterization of Screen-printed ex-Gr Conductor

**Fig. 3:**
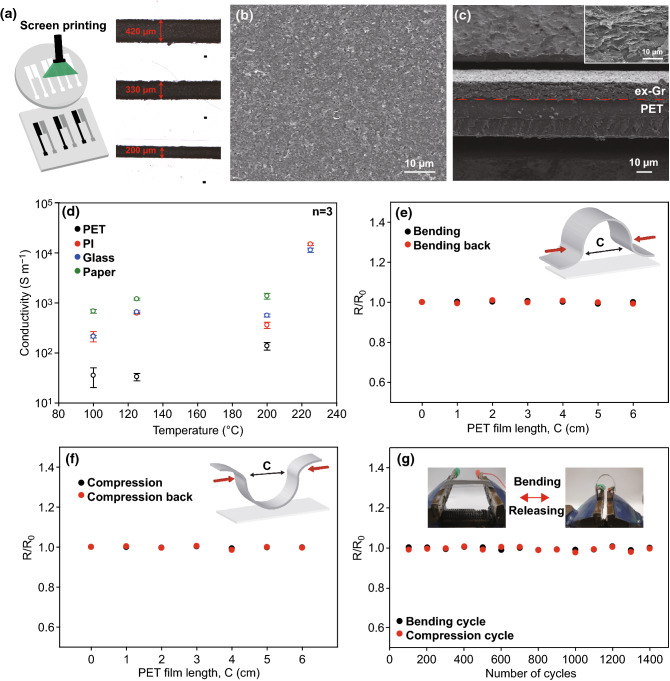
**a** A schematic of screen-printing process using ex-Gr ink and optical microscope images of printed ex-Gr conductors at different resolutions (200, 330, and 420 µm). **b** Surface and **c** cross-sectional SEM images of printed ex-Gr conductor (Inset: high-resolution SEM image of printed ex-Gr conductor). **d** Electrical conductivity of printed ex-Gr conductor (PET, PI, glass, and paper). **e** Bending and **f** compressing tests for evaluating resistance of different bending length. **g** Fatigue test for evaluating resistance of bending over 1400 cycles

The highly concentrated and conductive ex-Gr dispersed in terpineol/ethanol solution could be readily used as a screen-printable conductive ink. Based on comparison of the TC flow-induced ink preparation and other conventional methods, the TC flow-induced process is faster and more efficient for obtaining a high concentration of ex-Gr inks (Table S1). It should also be noted that the ultrahigh concentration (> 47.5 mg mL^−1^) of the ex-Gr ink allowed us to eliminate the post-evaporation step and the addition of binders often required for the screen-printing process. Figure 3a shows screen-printed ex-Gr lines with widths of 420, 330, and 200 µm. The plane-view SEM image of the printed lines (Fig. 3b) displays highly compact and interconnected networks of ex-Gr flakes, which is favorable for high electron transfer. The cross-sectional SEM image in Fig. 3c shows a good adhesion at the interface of the printed ex-Gr and PET substrate. A small inner volume is observed between the ex-Gr layers (Inset of Fig. 3c). To minimize the influence of the inner volume on the electrical and mechanical properties of the printed ex-Gr conductor, the annealing process was performed on a screen-printed ex-Gr conductor. The conductivity of the printed ex-Gr conductor was significantly influenced by the annealing temperature and film thickness (Figs. 3d and S3). The thermal annealing is a very efficient method to improve the conductivity of a graphene conductor, since it removes the impurities and solvents. In addition, degradation of binders occurs during annealing, resulting in a compactly interconnected graphene layer structure, which is responsible for high conductivity of the ex-Gr conductor [69]. When the annealing temperature was increased from 100 to 225 °C, the conductivity increased from 2.17 × 10^2^ to 1.49 × 10^4^ S m^−1^. The conductivity of the ex-Gr conductor based on a thermal annealing process is superior or comparable to other previous techniques, such as plasma and thermal annealing (Table S3). In addition, as the thickness decreased from 41 to 20 µm, the conductivity increased from 2.59 × 10^3^ to 1.49 × 10^4^ S m^−1^. Basis on these results, a high conductivity of 1.49 × 10^4^ S m^−1^ was achieved at an annealing temperature of 225 °C and an ex-Gr film thickness of 20 µm. This conductivity value for Gr ink is superior to other previously reported values for graphene-based conductors even with higher concentrations of graphene (Table S2). Further, the mechanical tolerance of the printed ex-Gr conductors on different substrates of PET, PI, and paper was investigated under bending and compressing. The change in the resistance of the ex-Gr conductor was measured at different bending and compressing states in forward and reverse directions (Figs. 3e, f and S4). A negligible change in the resistance was observed in these measurements for all printed ex-Gr conductors. The resistance of the ex-Gr electrode was further measured during 1400 bending‒releasing and compressing‒releasing cycles (Figs. 3g and S4). The resistance remained stable, and the initial resistance was almost maintained during fatigue tests. Observation of SEM images revealed that the ex-Gr conductor has a good adhesion at the interface of electrode and substrate with no mechanical damages after bending and fatigue tests (Fig. S5). The resistance tests demonstrate the combined properties of the ex-Gr conductor, i.e., its high conductivity and high mechanical stability.

### Electrochemical Performance of Screen-Printed Na^+^ Sensor

**Fig. 4:**
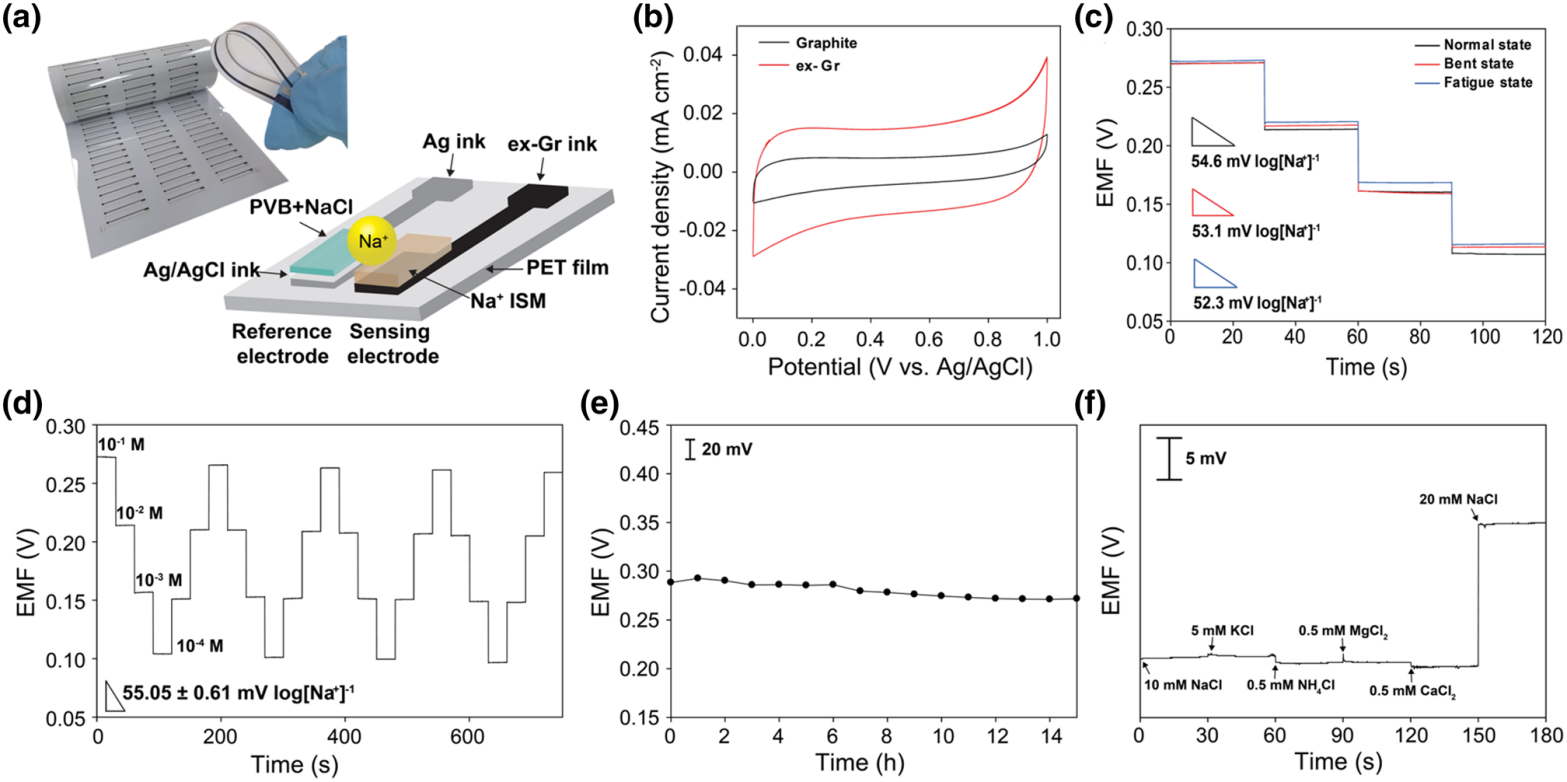
**a** A photograph of screen-printed Na^+^ sensors and a schematic of Na^+^-sensor structure. **b** CV curves of printed graphite and ex-Gr electrodes measured at a scan rate of 50 mV s^−1^ in 10^−1^ M NaCl. **c** EMF responses of Na^+^ sensors measured in the 10^−1^‒10^−4^ M range of NaCl concentration under mechanically normal and bent states and after fatigue test of 1400 bending and releasing cycles. **d** Repeatability test of Na^+^ sensor in various NaCl concentration solutions. **e** Long-term stability of Na^+^ sensor measured for 15 h in 10^−1^ M NaCl solution. **f** Selectivity test of Na^+^, K^+^, NH_4_^+^, Ca^2+^, Mg^2+^ ions

The highly conductive and flexible ex-Gr conductor can be used to fabricate flexible screen-printed electrochemical ion sensors. As an electrochemical ion sensor, a sodium ion selective membrane (Na^+^-ISM), used to diagnose hyponatremia, was selected for the proof-of-concept testing. Ex-Gr and Ag inks were printed using a custom-designed stencil mask onto a highly flexible and thin PET substrate (Fig. 4a). The Na^+^-ISM was coated on the surface of the printed ex-Gr electrode serving as the sensing electrode. For a reference electrode, the Ag/AgCl ink was printed onto the reaction part of the Ag circuit, followed by coating sodium chloride-containing polyvinyl butyral on the Ag/AgCl-coated surface of the reference electrode. The cross section of the Na^+^ sensor based on the two-electrode configuration is illustrated in Fig. 4a. It is worth noting that owing to the high surface area and electrical double-layer capacitance, the ex-Gr coating serves as an internal ion-to-electron transducer (also known as a solid contact) that facilitates the potentiometric performance. Compared to the graphite ink-based electrode, the ex-Gr electrode shows superior electrical double-layer capacitance and potential stability (Figs. 4b and S6). In addition, the ex-Gr electrode strongly resists the formation of a water layer between the ISM and electrode and is adequately insensitive to gas species (Fig. S7).

The potentiometric performance of the Na^+^ sensor was evaluated by measuring the electromotive force (EMF) between the Na^+^-ISM and Ag/AgCl electrodes using various NaCl solutions at physiologically relevant concentrations. The calibration curve of the EMF versus Na^+^ concentration is also depicted in Fig. S8. The Na^+^ sensor shows an average sensitivity of 54.0 mV per decade of Na^+^ concentration (*n* = 5) with a relative standard deviation (RSD) of 1.2%. This average sensitivity indicates the near-Nernstian behavior of the device. The small standard deviation of the slope (± 0.65 mV log[Na^+^]^−1^) and absolute potential (± 7.9 mV) from five sensors demonstrate the good reproducibility of the Na^+^ sensor fabricated by the screen-printing process. The Na^+^ sensor exhibits a rapid response time of 3.6 s, as measured by successively increasing the Na^+^ concentration from 100 to 1 mM (Fig. S9). This fast response time is important for the real-time monitoring of sweat, because the sensor would allow quick and accurate assessment of changes in the Na^+^ concentration. Based on the intersection of two slopes of EMF versus concentration sodium ion (Fig. S10), the detection limit of the Na^+^ sensor was calculated to be 14.8 µM. The effect of annealing temperature on the performance of the Na^+^ sensor was investigated by measuring the sensitivity with different annealing temperatures of 60, 100, 125, and 200 °C (Fig. S11). The Na^+^ sensors exhibited an average sensitivity of 53.1 ± 2.7 mV log[Na^+^]^−1^. The small standard deviation indicates no effect of annealing temperature on the electrochemical sensing performance of the Na^+^ sensor. The sensitivity of the Na^+^ sensor was further investigated in a mechanically bent state (Fig. 4c). Remarkably, the Na^+^ sensor exhibits a slope of 53.1 mV log[Na^+^]^‒1^ (*R*^2^ = 0.9977), which is consistent with the response of the same sensor in the normal state. After a fatigue test of 1,400 bending and releasing cycles, a high sensitivity of 52.3 mV log[Na^+^]^‒1^ (*R*^2^ = 0.9999) for the Na^+^ sensor was maintained (Fig. 4c). This high sensing performance of the Na^+^ sensor against mechanical stress is attributed to the unique mechanical strength of the ex-Gr sheets and EC binder. The electrochemical stability of the Na^+^ sensor was also evaluated by a repeatability test. Figure 4d shows the dynamic EMF response of the Na^+^ sensor under the repeated sequential loading of different NaCl solutions in the concentration range 0.1‒100 mM. During this measurement, the original EMF responses of the Na^+^ sensor were retained without any hysteresis effects. After five consecutive repetitions, the Na^+^ sensor exhibited a sensitivity of 54.4 mV log[Na^+^]^‒1^, which is very similar to the initial value of 56.1 mV log[Na^+^]^‒1^, indicating excellent repeatability. To demonstrate reusability, the sensitivity of the Na^+^ sensor was evaluated repeatedly by measuring EMF responses and then washing with DI water. After 10 cycles of repeating tests, the Na^+^ sensor still exhibited a high sensitivity of 56.3 mV log[Na^+^]^‒1^, which is very close to the initial sensitivity of 55.4 mV log[Na^+^]^‒1^ (Fig. S12). This indicates good reusability of the Na^+^ sensor.

Further, potentiometric measurements were performed to investigate the durability and selectivity of the Na^+^ sensor. Its durability was investigated by evaluating the potential drift over a period of 15 h (Fig. 4e). The Na^+^ sensor exhibits a potential drift of 1.2 mV h^‒1^, which is considerably lower than that of other Na^+^ sensors based on other carbon or gold electrodes [70–72]. The selectivity of the Na^+^ sensor was investigated by determining the selectivity coefficients of the Na^+^ sensor for different interfering ions using the separate-solution method. The selectivity coefficients for K^+^, NH_4_^+^, Ca^2+^, and Mg^2+^ were found to be less than 1 (Table S4), indicating good ion selectivity of the Na^+^ sensor. To further demonstrate high selectivity of the Na^+^ sensor, the change in EMF response was measured by adding interfering ions such as 5 mM KCl, 0.5 mM NH_4_Cl, 0.5 mM MgCl_2_, and 0.5 mM CaCl_2_ to a 10 mM NaCl solution (Fig. 4f). The interfering ion concentrations were selected considering physiological concentrations in human sweat. The sensor showed no response to the addition of such ions. After the addition of a 10 mM NaCl solution, the EMF signal increased according to Nernstian behavior. To further investigate the effect of pH on the sensitivity of the Na^+^ sensor, the sensitivity was evaluated by measuring EMF response at different pH values of 4.50, 6.24, and 8.53, decreasing the concentration of the Na^+^ solution (Fig. S13). The EMF signals and sensitivities almost overlapped at the pH levels, indicating no interference of pH with the sensor performance of the Na^+^ sensor.

### On-Body Test of Sodium Ion Sensor

**Fig. 5:**
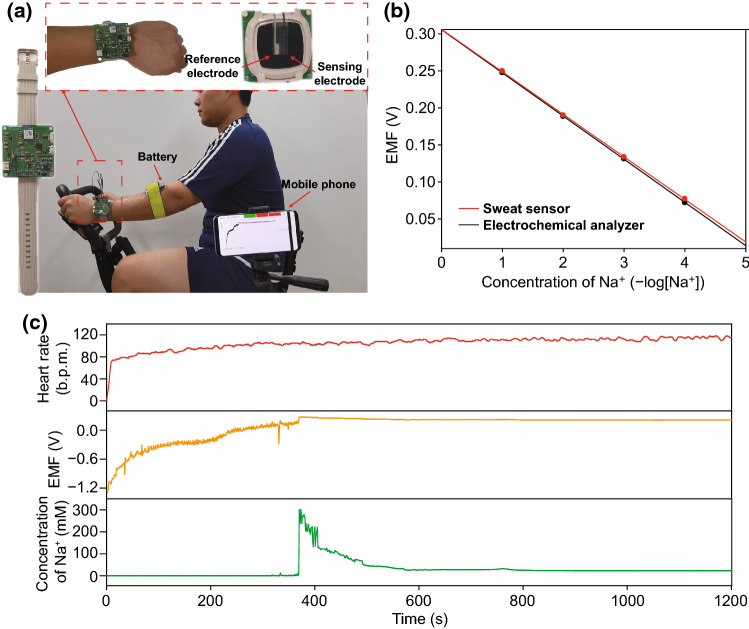
**a** A photographs of wearing wristwatch-type wearable device for real-time analysis during stationary cycling. **b** Calibration curves of electrochemical analyzer and sweat sensor. **c** Subject's simultaneous heart rate, EMF and converted concentration of Na^+^ during stationary cycling

To demonstrate real-time monitoring of sodium ions in human sweat with a screen-printed Na^+^ sensor based on ex-Gr ink, a wristwatch-type wearable device was fabricated by connecting the Na^+^ sensor and a PCB containing a Bluetooth module (Fig. 5a). The data collected continuously from the PCB were wirelessly transmitted to a smartphone application. To ensure accurate signal collection by the on-body test, the EMF-to-concentration calibration of the sweat sensor was confirmed before and after the on-body measurements (Fig. 5b). Figure 5c shows the on-body test condition with the subject wearing the experimental sweat watch sensor while riding a stationary exercise bike at room temperature (Movie S1). The maximum exercise power was 700 W and the total exercise time was 20 min, and the average heart rate of the subject was 108 bpm. The profiles of EMF and Na^+^ concentration collected during the real-time on-body sweat test are displayed in Fig. 5c. The collected EMF signals were converted to Na^+^ concentration based on a calibration curve obtained before the on-body test. When the subject started cycling, there was significant signal noise observed due to an insufficient amount of sweat for a signal from the two-electrode configuration. After approximately 370 s, EMF signals and corresponding Na^+^ concentrations rapidly changed, indicating that there was sufficient sweat collected for the two-electrode sensor. This phenomenon was also observed for previously reported electrochemical ion sensors [73–76] and is related to the perspiration rate of the subject while exercising. The Na^+^ concentration gradually stabilized, reaching approximately 24 mM, which is the physiological Na^+^ ion concentration. The resultant real-time data of the Na^+^ sensor are similar to those obtained in previously reported on-body tests [71, 77–79].

## Conclusions

We have successfully demonstrated a scalable and efficient fluid dynamics process for the preparation of high-concentration conductive inks from defect-free ex-Gr with high electrical conductivity. To date, the preparation of graphene-based inks has suffered from a lack of mass production and a low concentration of graphene suspensions. The TC flow-induced process enabled an efficient exfoliation of graphite into defect-free graphene nanosheets in a high yield of 53.5% and a high concentration of 47.5 mg mL^−1^, which are superior to other solution-based methods. The ultrahigh concentration of the ex-Gr ink allowed us to eliminate the post-evaporation step and additional binders that are often required for the screen-printing process. The maximum graphite loading was 200 mg mL^−1^ for the fluid dynamics process owing to the high viscosity of the graphite loading in the limited space of our fluidic reactor. However, the fluidic reactor can be easily scaled up, and thus, it is possible that a larger fluidic reactor can process graphite loading over 200 mg mL^−1^. The as-obtained ex-Gr ink facilitated the screen-printing process, which was used to fabricate a high-performance electro-mechanical ex-Gr conductor. The ex-Gr conductor exhibited a high electrical conductivity of 1.49 × 10^4^ S m^−1^ and low resistance variation in mechanical bending and fatigue tests. Using the ex-Gr ink with these attractive merits, potentiometric Na^+^ sensors based on a two-electrode configuration were fabricated. The Na^+^ sensors showed rapid response times and high sensitivities in the mechanically normal and bent states with good repeatability and selectivity. Furthermore, a wristwatch-type Na^+^ sensor was fabricated by integrating it with a wireless electronic module. A successful on-body test was performed by the real-time wireless monitoring of the Na^+^ concentration of the sweat of a volunteer during an indoor cycling exercise. The wearable sweat sensor could be further improved by integrating temperature and other ion sensors (e.g., K^+^, pH, and Cl^−^) to analyze multiple analytes in sweat, and thus for real-time monitoring of the physiological status of individuals. The highly conductive and highly concentrated graphene ink presented in this work opens a promising opportunity for the fabrication of low-cost, scalable, and high-performance electrochemical ion sensors for the practical real-time monitoring of human health.

## Supplementary Information

Below is the link to the electronic supplementary material.

Supplementary file1 (PDF 1238 KB)

Supplementary file2 (MP4 18816 KB)

## References

[1] Teymourian H, Parrilla M, Sempionatto JR, Montiel NF, Barfidokht A (2020). Wearable electrochemical sensors for the monitoring and screening of drugs. ACS Sen..

[2] Kim J, Campbell AS, Ávila BE-F, Wang J (2019). Wearable biosensors for healthcare monitoring. Nat. Biotechnol..

[3] Song Y, Min J, Gao W (2019). Wearable and implantable electronics: moving toward precision therapy. ACS Nano.

[4] Min J, Sempionatto JR, Teymourian H, Wang J, Gao W (2021). Wearable electrochemical biosensors in north america. Biosens. Bioelectron..

[5] Bariya M, Li L, Ghattamaneni R, Ahn CH, Nyein HYY (2020). Glove-based sensors for multimodal monitoring of natural sweat. Sci. Adv..

[6] Ling Y, An T, Yap LW, Zhu B, Gong S (2020). Disruptive, soft, wearable sensors. Adv. Mater..

[7] Yu Y, Nyein HYY, Gao W, Javey A (2020). Flexible electrochemical bioelectronics: the rise of in situ bioanalysis. Adv. Mater..

[8] Li T, Li Y, Zhang T (2019). Materials, structures, and functions for flexible and stretchable biomimetic sensors. Acc. Chem. Res..

[9] Lou Z, Wang L, Jiang K, Wei Z, Shen G (2020). Reviews of wearable healthcare systems: materials, devices and system integration. Mater. Sci. Eng. R Rep..

[10] Shen Y, Wang Y, Luo Z, Wang B (2020). Durable, sensitive, and wide-range wearable pressure sensors based on wavy-structured flexible conductive composite film. Macromol. Mater. Eng..

[11] Yun J, Lee H, Song C, Jeong YR, Park JW (2020). A fractal-designed stretchable and transparent microsupercapacitor as a skin-attachable energy storage device. Chem. Eng. J..

[12] Chu T, Chu J, Gao B, He B (2020). Modern evolution of paper-based analytical devices for wearable use: from disorder to order. Analyst.

[13] Lee HC, Hsieh EY, Yong K, Nam S (2020). Multiaxially-stretchable kirigami-patterned mesh design for graphene sensor devices. Nano Res..

[14] Wang S, Bai Y, Yang X, Liu L, Li L (2020). Highly stretchable potentiometric ion sensor based on surface strain redistributed fiber for sweat monitoring. Talanta.

[15] Teymourian H, Barfidokht A, Wang J (2020). Electrochemical glucose sensors in diabetes management: an updated review (2010–2020). Chem. Soc. Rev..

[16] Sreenilayam SP, Ahad IU, Nicolosi V, Garzon VA, Brabazon D (2020). Advanced materials of printed wearables for physiological parameter monitoring. Mater. Today.

[17] Lee H, Hong YJ, Baik S, Hyeon T, Kim D-H (2018). Enzyme-based glucose sensor: from invasive to wearable device. Adv. Healthc. Mater..

[18] Yan Y, Gao W (2019). Wearable and flexible electronics for continuous molecular monitoring. Chem. Soc. Rev..

[19] Bandodkar AJ, Wang J (2014). Non-invasive wearable electrochemical sensors—a review. Trends Biotechnol..

[20] Arnold MA, Small GW (2005). Noninvasive glucose sensing. Anal. Chem..

[21] Heikenfeld J, Jajack A, Rogers J, Gutruf P, Tian L (2018). Wearable sensors: modalities, challenges, and prospects. Lab Chip.

[22] Torrente-Rodríguez RM, Tu J, Yang Y, Min J, Wang M (2020). Investigation of cortisol dynamics in human sweat using a graphene-based wireless mHealth system. Matter.

[23] Xu J, Zhang Z, Gan S, Gao H, Kong H (2020). Highly-stretchable fiber-based potentiometric ion sensors for multichannel real-time analysis of human sweat. ACS Sens..

[24] An Q, Gan S, Xu J, Bao Y, Wu T (2019). A multichannel electrochemical all-solid-state wearable potentiometric sensor for real-time sweat ion monitoring. Electrochem. Commun..

[25] Nyein HYY, Bariya M, Kivimäki L, Uusitalo S, Liaw TS (2019). Regional and correlative sweat analysis using high-throughput microfluidic sensing patches toward decoding sweat. Sci. Adv..

[26] Nyein HYY, Tai L-C, Ngo QP, Chao M, Zhang GB (2018). A wearable microfluidic sensing patch for dynamic sweat secretion analysis. ACS Sens..

[27] Zhang S, Zahed MA, Sharifuzzaman Md, Yoon S, Hui X (2021). A wearable battery-free wireless and skin-interfaced microfluidics integrated electrochemical sensing patch for on-site biomarkers monitoring in human perspiration. Biosens. Bioelectron..

[28] Ochoa M, Rahimi R, Zhou J, Jiang H, Yoon CK (2020). Integrated sensing and delivery of oxygen for next-generation smart wound dressings. Microsyst. Nanoeng..

[29] Bobovych S, Sayeed F, Banerjee N, Robucci R, Allen RP (2020). RestEaZe: low-power accurate sleep monitoring using a wearable multi-sensor ankle band. Smart Health.

[30] Manjakkal L, Dang W, Yogeswaran N, Dahiya R (2019). Textile-based potentiometric electrochemical pH sensor for wearable applications. Biosensors.

[31] Hatamie A, Angizi S, Kumar S, Pandey CM, Simchi A (2020). Textile based chemical and physical sensors for healthcare monitoring. J. Electrochem. Soc..

[32] He W, Wang C, Wang H, Jian M, Lu W (2019). Integrated textile sensor patch for real-time and multiplex sweat analysis. Sci. Adv..

[33] Bandodkar AJ, Jia W, Yardimcı C, Wang X, Ramirez J (2015). Tattoo-based noninvasive glucose monitoring: a proof-of-concept study. Anal. Chem..

[34] Sempionatto JR, Jeerapan I, Krishnan S, Wang J (2020). Wearable chemical sensors: emerging systems for on-body analytical chemistry. Anal. Chem..

[35] Bandodkar AJ, Jeerapan I, Wang J (2016). Wearable chemical sensors: present challenges and future prospects. ACS Sens..

[36] Ray TR, Choi J, Bandodkar AJ, Krishnan S, Gutruf P (2019). Bio-integrated wearable systems: a comprehensive review. Chem. Rev..

[37] Li Q, Zhang J, Li Q, Li G, Tian X (2019). Review of printed electrodes for flexible devices. Front. Mater..

[38] Sfragano PS, Laschi S, Palchetti I (2020). Sustainable printed electrochemical platforms for greener analytics. Front. Chem..

[39] Bonaccorso F, Bartolotta A, Coleman JN, Backes C (2016). 2D-crystal-based functional inks. Adv. Mater..

[40] Hu G, Kang J, Ng LWT, Zhu X, Howe RCT (2018). Functional inks and printing of two-dimensional materials. Chem. Soc. Rev..

[41] Tran TS, Dutta NK, Choudhury NR (2018). Graphene inks for printed flexible electronics: graphene dispersions, ink formulations, printing techniques and applications. Adv. Colloid Interface Sci..

[42] Pan K, Fan Y, Leng T, Li J, Xin Z (2018). Sustainable production of highly conductive multilayer graphene ink for wireless connectivity and IoT applications. Nat. Commun..

[43] Park S, Ruoff RS (2009). Chemical methods for the production of graphenes. Nat. Nanotechnol..

[44] Nicolosi V, Chhowalla M, Kanatzidis MG, Strano MS, Coleman JN (2013). Liquid exfoliation of layered materials. Science.

[45] Mao S, Pu H, Chen J (2012). Graphene oxide and its reduction: modeling and experimental progress. RSC Adv..

[46] Paton KR, Varrla E, Backes C, Smith RJ, Khan U (2014). Scalable production of large quantities of defect-free few-layer graphene by shear exfoliation in liquids. Nat. Mater..

[47] Bellani S, Petroni E, Del Rio Castillo AE, Curreli N, Martín-García B (2019). Scalable production of graphene inks via wet-jet milling exfoliation for screen-printed micro-supercapacitors. Adv. Funct. Mater..

[48] Shin Y, Vranic S, Just-Baringo X, Gali SM, Kisby T (2020). Stable, concentrated, biocompatible, and defect-free graphene dispersions with positive charge. Nanoscale.

[49] Tehrani F, Beltrán-Gastélum M, Sheth K, Karajic A, Yin L (2019). Laser-induced graphene composites for printed, stretchable, and wearable electronics. Adv. Mater. Technol..

[50] Wei N, Yu L, Sun Z, Song Y, Wang M (2019). Scalable salt-templated synthesis of nitrogen-doped graphene nanosheets toward printable energy storage. ACS Nano.

[51] Yi M, Shen Z (2015). A review on mechanical exfoliation for the scalable production of graphene. J. Mater. Chem. A.

[52] Lotya M, King PJ, Khan U, De S, Coleman JN (2010). High-concentration, surfactant-stabilized graphene dispersions. ACS Nano.

[53] Torrisi F, Hasan T, Wu W, Sun Z, Lombardo A (2012). Inkjet-printed graphene electronics. ACS Nano.

[54] Tyurnina AV, Tzanakis I, Morton J, Mi J, Porfyrakis K (2020). Ultrasonic exfoliation of graphene in water: a key parameter study. Carbon.

[55] Manna K, Wang L, Loh KJ, Chiang W-H (2019). Printed strain sensors using graphene nanosheets prepared by water-assisted liquid phase exfoliation. Adv. Mater. Interfaces.

[56] Tran TS, Park SJ, Yoo SS, Lee T-R, Kim T (2016). High shear-induced exfoliation of graphite into high quality graphene by Taylor–Couette flow. RSC Adv..

[57] Jeong J-M, Jin SB, Park HJ, Park SH, Jeon H (2020). Large-scale fast fluid dynamic processes for the syntheses of 2D nanohybrids of metal nanoparticle-deposited boron nitride nanosheet and their glycolysis of poly(ethylene terephthalate). Adv. Mater. Interfaces.

[58] Yoon JH, Kim S-M, Eom Y, Koo JM, Cho H-W (2019). Extremely fast self-healable bio-based supramolecular polymer for wearable real-time sweat-monitoring sensor. ACS Appl. Mater. Interfaces.

[59] Yoon JH, Kim S-M, Park HJ, Kim YK, Oh DX (2020). Highly self-healable and flexible cable-type pH sensors for real-time monitoring of human fluids. Biosnes. Bioelectron..

[60] Jeong J-M, Kang HG, Kim H-J, Hong SB, Jeon H (2018). Hydraulic power manufacturing for highly scalable and stable 2D nanosheet dispersions and their film electrode application. Adv. Funct. Mater..

[61] Robinson JA, Puls CP, Staley NE, Stitt JP, Fanton MA (2009). Raman topography and strain uniformity of large-area epitaxial graphene. Nano Lett..

[62] Hao Y, Wang Y, Wang L, Ni Z, Wang Z (2010). Probing layer number and stacking order of few-layer graphene by raman spectroscopy. Small.

[63] Gomez CV, Tene T, Guevara M, Usca GT, Colcha D (2019). Preparation of few-layer graphene dispersions from hydrothermally expanded graphite. Appl. Sci..

[64] Karim N, Zhang M, Afroj S, Koncherry V, Potluri P (2018). Graphene-based surface heater for de-icing applications. RSC Adv..

[65] Afroj S, Tan S, Abdelkader AM, Novoselov KS, Karim N (2020). Highly conductive, scalable, and machine washable graphene-based e-textiles for multifunctional wearable electronic applications. Adv. Funct. Mater..

[66] Jeon H, Jeong J-M, Kang HG, Kim H-J, Park J (2018). Scalable water-based production of highly conductive 2D nanosheets with ultrahigh volumetric capacitance and rate capability. Adv. Energy Mater..

[67] Hong SB, Jeong J-M, Kang HG, Seo D, Cha Y (2018). Fast and scalable hydrodynamic synthesis of MnO_2_/defect-free graphene nanocomposites with high rate capability and long cycle life. ACS Appl. Mater. Interfaces.

[68] Alfano B, Polichetti T, Mauriello M, Miglietta ML, Ricciardella F (2016). Modulating the sensing properties of graphene through an eco-friendly metal-decoration process. Sens. Actuat. B.

[69] Arapov K, Bex G, Hendriks R, Rubingh E, Abbel R (2016). Conductivity enhancement of binder-based graphene inks by photonic annealing and subsequent compression rolling. Adv. Eng. Mater..

[70] Roy S, David-Pur M, Hanein Y (2017). Carbon nanotube-based ion selective sensors for wearable applications. ACS Appl. Mater. Interfaces.

[71] Bandodkar AJ, Molinnus D, Mirza O, Guinovart T, Windmiller JR (2014). Epidermal tattoo potentiometric sodium sensors with wireless signal transduction for continuous non-invasive sweat monitoring. Biosens. Bioelectron..

[72] Gao W, Emaminejad S, Nyein HYY, Challa S, Chen K (2016). Fully integrated wearable sensor arrays for multiplexed in situ perspiration analysis. Nature.

[73] Alizadeh A, Burns A, Lenigk R, Gettings R, Ashe J (2018). A wearable patch for continuous monitoring of sweat electrolytes during exertion. Lab Chip.

[74] McCaul M, Porter A, Barrett R, White P, Stroiescu F (2018). Wearable platform for real-time monitoring of sodium in sweat. ChemPhysChem.

[75] Liu G, Ho C, Slappey N, Zhou Z, Snelgrove SE (2016). A wearable conductivity sensor for wireless real-time sweat monitoring. Sens. Actuat. B.

[76] Ma B, Chi J, Xu C, Ni Y, Zhao C (2020). Wearable capillary microfluidics for continuous perspiration sensing. Talanta.

[77] Parrilla M, Ortiz-Gómez I, Cánovas R, Salinas-Castillo A, Cuartero M (2019). Wearable potentiometric ion patch for on-body electrolyte monitoring in sweat: toward a validation strategy to ensure physiological relevance. Anal. Chem..

[78] Glennon T, O'Quigley C, McCaul M, Matzeu G, Beirne S (2016). 'Sweatch': a wearable platform for harvesting and analysing sweat sodium content. Electroanalysis.

[79] Matzeu G, O'Quigley C, McNamara E, Zuliani C, Fay C (2016). An integrated sensing and wireless communications platform for sensing sodium in sweat. Anal. Methods.

