# Supplementary material for: Highly Concentrated, Conductive, Defect-free Graphene Ink for Screen-Printed Sensor Application
Source: Nanomicro Lett. 2021 Mar 8;13:87. doi: 10.1007/s40820-021-00617-3 (PMC8006523; doi:10.1007/s40820-021-00617-3)
Supplement: Supplementary file 1 — Supplementary file1 (PDF 1238 KB) [file 40820_2021_617_MOESM1_ESM.pdf]

Supporting Information for

Highly Concentrated, Conductive, Defect-free Graphene Ink for Screen-Printed Sensor Application

Dong Seok Kim[1, #], Jae-Min Jeong[2, #], Hong Jun Park[1, #], Yeong Kyun Kim,[1] Kyoung G. Lee[3, *], Bong Gill Choi[1, *]

[1]Department of Chemical Engineering, Kangwon National University, Samcheok, Gangwon–do 25913, Republic of Korea

[2]Resources Utilization Research Center, Korea Institute of Geoscience and Mineral Resources, Daejeon 34132, Republic of Korea

[3]Nano-Bio Application Team, National Nanofab Center, Daejeon 34141, Republic of Korea

#Dong Seok Kim, Jae-Min Jeong, and Hong Jun Park contributed equally to this work

*Corresponding authors. E-mail: kglee@nnfc.re.kr (K.G.L), bgchoi@kangwon.ac.kr (B.G.C)

Supplementary Tables and Figures

Table 1 Comparison of fluid dynamic process and other previously reported solution-based methods in terms of exfoliation performance

Method	Solvent	Time	Yield	Concentration	I_D/I_G	Refs.
Shear exfoliation	NMP	4 h	~3.35%	~0.07 mg/mL	0.18	[S1]
Blender	Anionic/nonionic surfactant	4 h	~1%	~1 mg/mL	0.3~	[S2]
high pressure homogenize	DMF	10 min	~2.68%	~0.084 mg/mL	High defect	[S3]
Bath sonication	Pluronic P-123 (aq.)	5 h	N/A	~1.5 mg/mL	0.1~	[S4]
Bath sonication	NMP	462 h	~4%	~1.2 mg/mL	N/A	[S5]
Tip ultrasonication	[Bmim][Tf_2N]	~1 h	N/A	~0.95 mg/mL	6~	[S6]
Tip ultrasonication	DNA (aq.)	~6 h	N/A	~2.29 mg/mL	0.61~	[S7]
Planetary mill	Ammonia borane (NH_3BH_3)	~4 h	~25%	N/A	0.5~	[S8]
Bath sonication	Sodium cholate (aq.)	~430 h	~2	~0.3 mg/mL	0.4	[S9]
**Fluid-dynamics Process**	**Terpineol/Ethanol with 10 wt% of EC**	**1 h**	**~53.5%**	**~47.5 mg/mL**	**0.21**	**This work**

NMP; *N*-methyl-2-pyrrolidone, DMF; Dimethylformamide, DNA; deoxyribonucleic acid, TPU; Thermoplastic polyurethane.

Table 2 Comparison of fluid dynamic process for ex-Gr ink and other previously reported solution-based methods in terms of preparation method, process time, printing method, and conductivity

Method	Formula (solvent, surfactant)	Time	Printing method	Conductivity [S/m]	Refs.
Sonication	Deionized water	2 h	Inkjet	8.74×10^2 (Only RGO)	[S10]
Ultrasonication	NMP	9 h	Inkjet	100 (Only graphene)	[S11]
Tip sonication	NMP	7 h	Inkjet	3×10^3 (Only graphene)	[S12]
Ultrasonication	0.1% (w/v) EC in cyclohexanone	30 min	Inkjet	9.24×10^3 (EC:Gr~1.5:8.5)	[S13]
Electrochemical	DMF	1 h	Inkjet	2.5×10^3 (Only GNPs)	[S14]
Ultrasonication	5 w/v% EC in toluene:ethanol	20-40 h	Inkjet	100 (EC:Gr~1.2:8.8)	[S15]
Ball milling	10 wt% PTFE in ethanol	1 h	Screen	13 (PTFE:GNPs/PANI=1:9)	[S16]
High-shear mixing	Copolymer in isopropanol	1 h	Screen	1.33×10^3 (Binder:Gr~7.5:2.5)	[S17]
Three-roll milling	75 wt% EC in isopropanol	1 h	Screen	1.7×10^3 (EC:Gr~2.3:7.7)	[S18]
**Fluid-dynamics Process**	**10 wt% EC in Terpineol/Ethanol**	**2 h**	**Screen**	1.49×10^4 **(EC:Gr~1:9)**	**This work**

NMP; *N*-methyl-2-pyrrolidone, EC; ethyl cellulose, DMF; *N*,*N*-dimethylformamide, PTFE; polytetrafluoroethylene, RGO; reduced graphene oxide, Gr; graphene, GNPs; graphene nanoplatelet.

Table 3 Comparison of our thermal annealing condition and other previously reported annealing processes

Materials	Solvent	Annealing Technique (Condition)	Conductivity [S/m]	Refs.
Graphene	DMF/terpineol	Thermal (400°C)	5×10^{-6}	[S15]
Reduced graphene oxide	Water, Etylene glycol	Plasma (138°C, Ar)	2[kΩ q^{-1}]	[S19]
Graphene	Acetone/Ethyl lactate	Thermal (100°C)	4×10^4	[S20]
Graphene	Water/Carboxymethyl cellulose sodium salt	Thermal (70°C)	4.23×10^4	[S21]

Graphene	2,3-butanediol/Isopropanol	Thermal (300℃)	8.7 × 10^3	[S22]
Graphene	NMP/Vinyl acetate/Isopropanol	Thermal (100℃)	4 × 10^4	[S23]
Graphene	NMP/Ethyl cellulose	Thermal (35℃)	6.5 × 10^{-10}	[S24]
Graphene	Ethylene cellulose/ethanol	Thermal (250℃)	2.5 × 10^4	[S25]
**Graphene**	**10 wt% EC in Terpineol/Ethanol**	**Thermal (225℃, Vacuum)**	**1.49 × 10^4**	**This work**

PVP; polyvinylpyrrolidone, NMP; *N*-methyl-2-pyrrolidone, DMF; *N*,*N*-dimethylformamide.

Table 4 Selectivity coefficients of various interfering ions for sodium sensor determined by SSM

Ion (J)	log K_{IJ}^{pot}	K_{IJ}^{pot}
Ca^{2+}	-5.890	1.29 × 10^{-6}
Mg^{2+}	-3.915	1.22 × 10^{-4}
K^+	-3.869	1.35 × 10^{-4}
H^+	-1.925	1.19 × 10^{-2}
NH_4^+	-5.142	7.20 × 10^{-6}

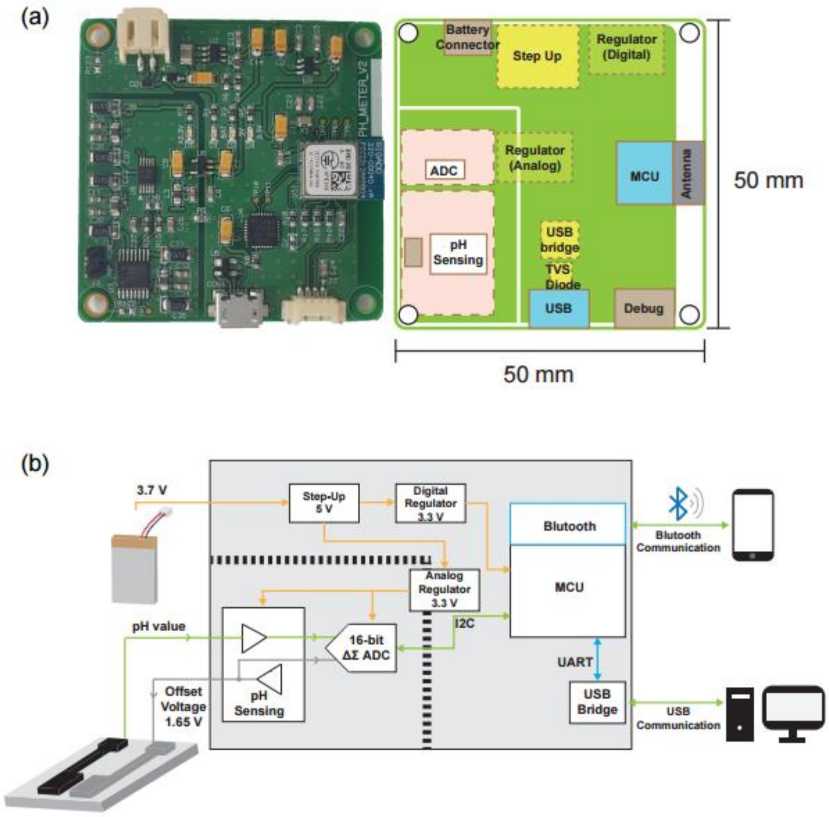

Fig. S1 a A photograph image and schematic of PCB. **b** A schematic diagram of the PCB system

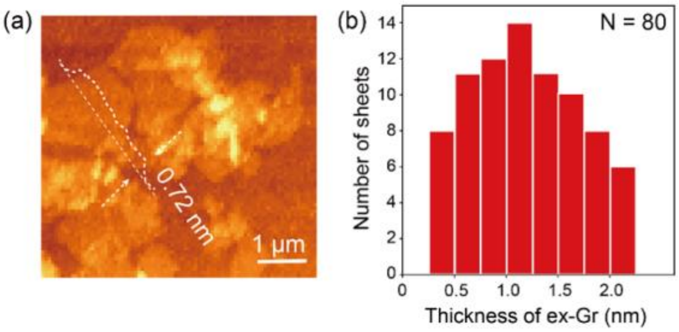

Fig. S2 a AFM image and **b** thickness distribution of ex-Gr

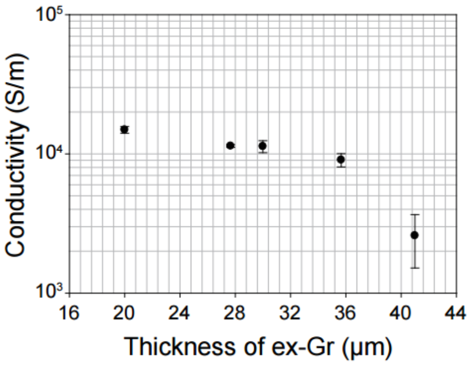

Fig. S3 Conductivity of ex-Gr conductor as a function of ex-Gr film thickness

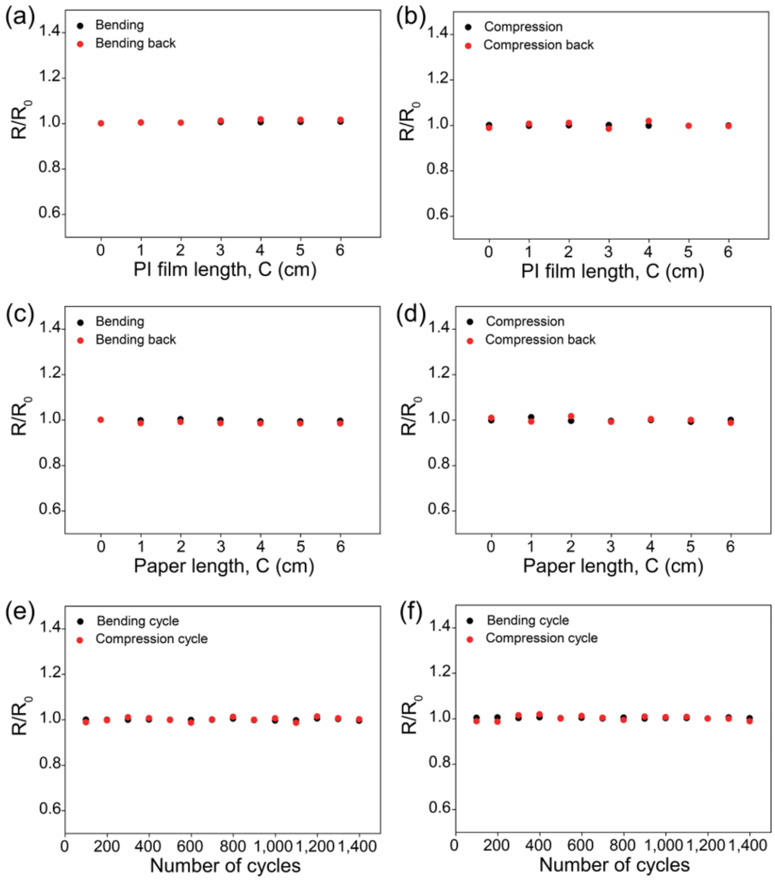

Fig. S4 Bending and compressing tests for evaluating resistance of printed ex-Gr conductors on PI film (**a** and **b**) and paper (**c** and **d**) substrates. Fatigue test for evaluating resistance of bending and compressing over 1,400 cycles for printed ex-Gr conductors on PI film (**e**) and paper (**f**) substrates

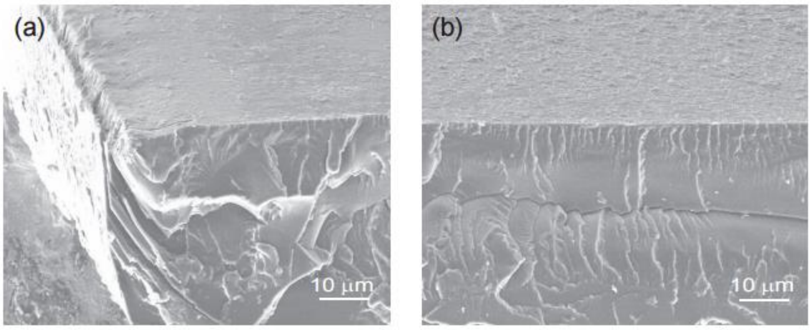

Fig. S5 SEM images of the ex-Gr conductor after **a** bending and **b** fatigue tests

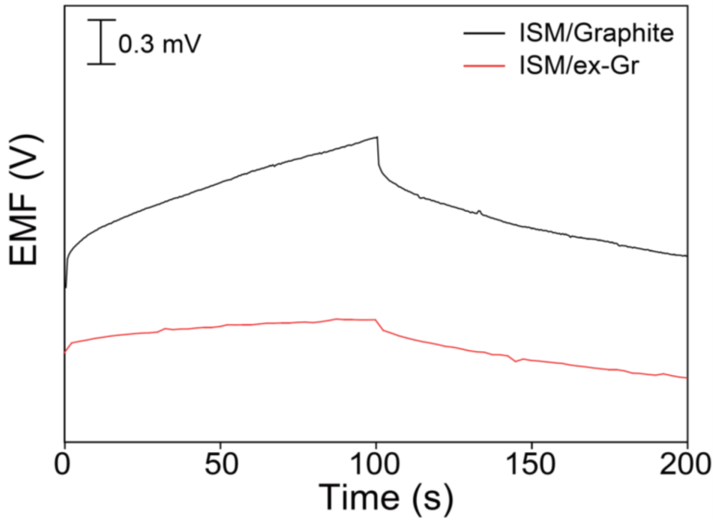

Fig. S6 Chronopotentiograms for electrical double-layer capacitance and potential stability of ISM/Graphite and ISM/ex-Gr electrodes in 0.1 M NaCl solution

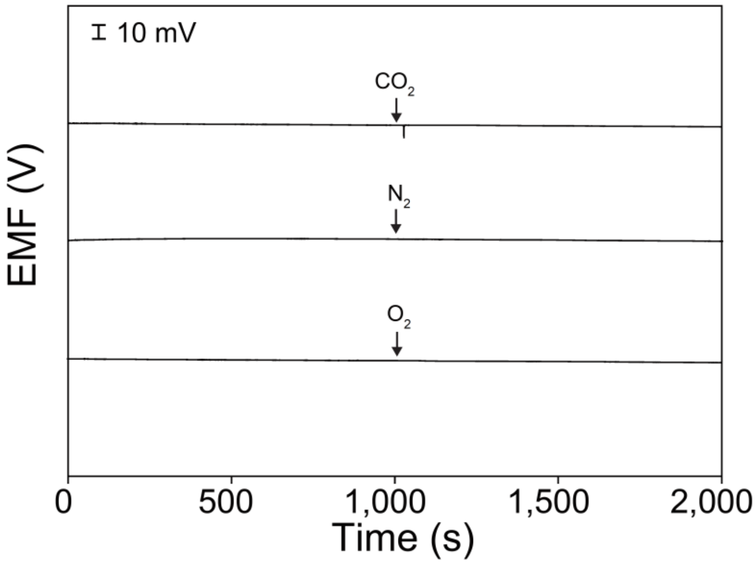

Fig. S7 Effect of potential stability of ISM/ex-Gr electrode at CO_2, N_2 and O_2 gas purging in 0.1 M NaCl solution

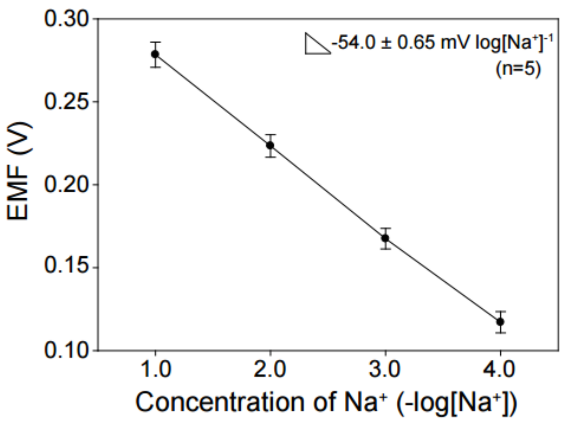

Fig. S8 Reproducibility calibration curves of Na^+ sensors (n=5)

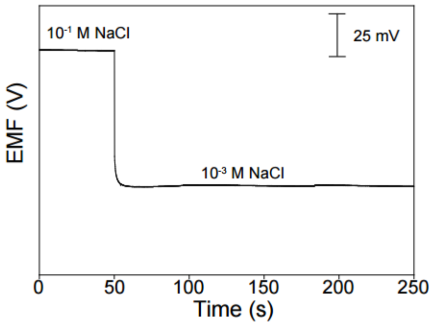

Fig. S9 Response time of Na^+ sensor by subsequently adding 10^{-3} M NaCl into 10^{-1} M NaCl solution and measuring the change of EMF signals

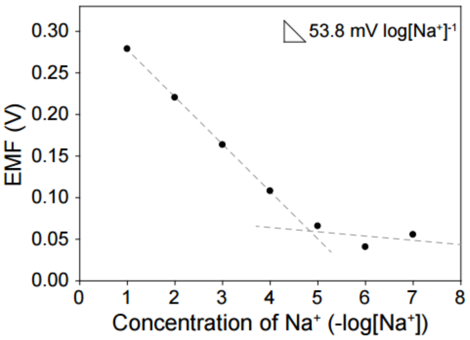

Fig. S10 Calibration curve for evaluating detection limit of Na^+ sensor

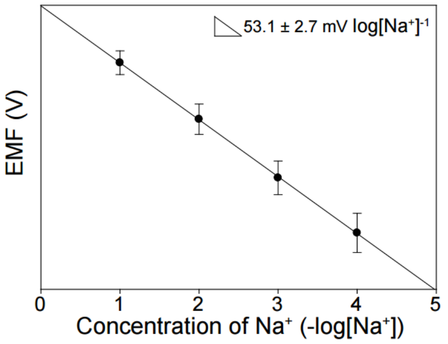

Fig. S11 Calibration curve of Na^+ sensors (n = 5) according to diluting concentration of NaCl solution at different annealing temperature of 60, 100, 125 and 200 ºC

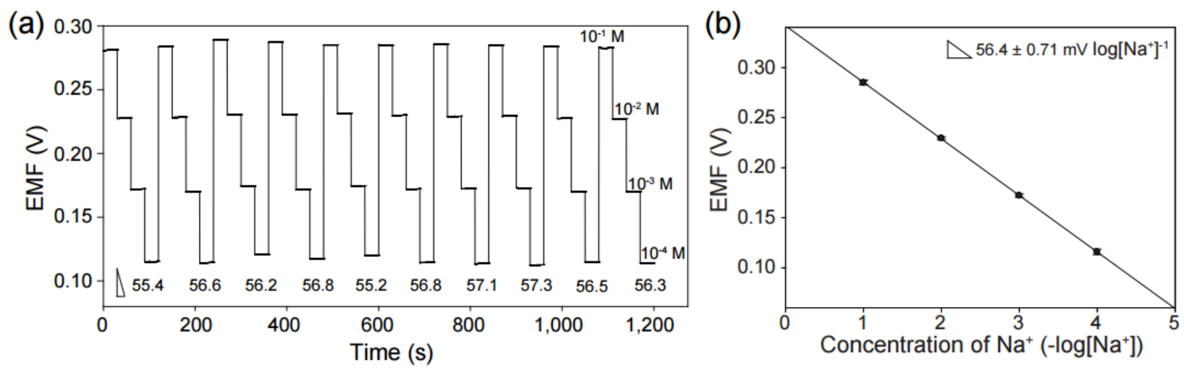

Fig. S12 a Reusability of Na^+ sensor by measuring EMF responses at 10^{-1}–10^{-4} M NaCl over 10 cycles with DI water washing at each cycle. **b** Calibration curves of Na^+ sensor obtained from reusability tests

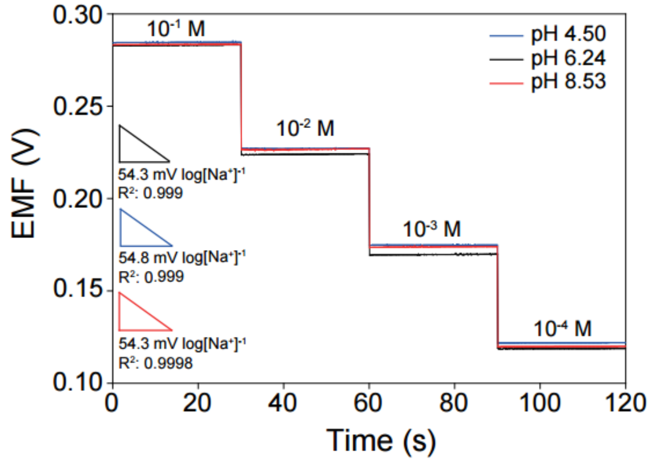

Fig. S13 EMF responses of Na^+ sensor at different pH values of 4.50, 6.24, and 8.53 with the decreasing concentration of the Na^+ solution from 10^{-1} to 10^{-4} M

Supplementary References

[S1] K. R. Paton, E. Varrla, C. Backes, R. J. Smith, U. Khan et al., Scalable production of large quantities of defect-free few-layer graphene by shear exfoliation in liquids. Nature Mater. **13**, 624 (2014). https://doi.org/10.1038/nmat3944

[S2] E. Varrla, K. R. Paton, C. Backes, A. Harvey, R. J. Smith et al., Turbulence-assisted shear exfoliation of graphene using household detergent and a kitchen blender. Nanoscale **6**, 11810 (2014). https://doi.org/10.1039/C4NR03560G

[S3] J. Shang, F. Xue, E. Ding, The facile fabrication of few-layer graphene and graphite nanosheets by high pressure homogenization. Chem. Commun. **51**, 15811 (2015). https://doi.org/10.1039/C5CC06151B

[S4] U. Khan, A. O’Neill, M. Lotya, S. De, J. N. Coleman, High-Concentration Solvent Exfoliation of Graphene. Small **6**, 864 (2010). https://doi.org/10.1002/smll.200902066

[S5] M. Uz, M. T. Lentner, K. Jackson, M. S. Donta, J. Jung et al., Fabrication of two-dimensional and three-dimensional high-resolution binder-free graphene circuits using a microfluidic approach for sensor applications. ACS Appl. Mater. Interfaces **12**, 13529 (2020). https://doi.org/10.1021/acsami.9b23460

[S6] P. He, J. Cao, H. Ding, C. Liu, J. Neilson et al., Screen-printing of a highly conductive graphene ink for flexible printed electronics. ACS Appl. Mater. Interfaces **11**, 32225 (2019). https://doi.org/10.1021/acsami.9b04589

[S7] D. Joseph, S. Seo, D. R. Williams, K. E. Geckeler, Double-stranded DNA-graphene hybrid: preparation and anti-proliferative activity. ACS Appl. Mater. Interfaces **6**, 3347 (2014). https://doi.org/10.1021/am405378x

[S8] L. Liu, Z. Xiong, D. Hu, G. Wu, P. Chen, Production of high quality single- or few-layered graphene by solid exfoliation of graphite in the presence of ammonia borane. Chem. Commun. **49**, 7890 (2013). https://doi.org/10.1039/C3CC43670E

[S9] M. Lotya, P. J. King, U.; Khan, S. De, J. N. Coleman, High-concentration, surfactant-stabilized graphene dispersions. ACS Nano **4**, 3155 (2014). https://doi.org/10.1021/nn1005304

[S10] L. Hauang, Y. Huang, J. Liang, X. Wan, Y. Chen, Graphene-based conducting inks for direct inkjet printing of flexible conductive patterns and their applications in electric circuits and chemical sensors. Nano Res. **4**, 675 (2011). https://doi.org/10.1007/s12274-011-0123-z

[S11] F. Torrisi, T. Hasan, W. Wu, Z. Sun, A. Lombardo et al., Inkjet-printed graphene electronics. ACS Nano **6**, 2992 (2012). https://doi.org/10.1021/nn2044609

[S12] D. J. Finn, M. Lotya, G. Cunningham, R. J. Smith, D. McCloskey et al., Inkjet deposition of liquid-exfoliated graphene and MoS_2 nanosheets for printed device applications. J. Mater. Chem. C **2**, 925 (2014). https://doi.org/10.1039/C3TC31993H

[S13] Y. Gao, W. Shi, W. Wang, Y. Leng, Y. Zhao, Inkjet printing patterns of highly conductive pristine graphene on flexible substrates. Ind. Eng. Chem. Res. **53**, 16777 (2014). https://doi.org/10.1021/ie502675z

[S14] F. Miao, S. Majee, M. Song, J. Zhao, S. Zhang et al., Inkjet printing of electrochemically-exfoliated graphene nano-platelets. Synth. Met. **220**, 318 (2016). https://doi.org/10.1016/j.synthmet.2016.06.029

[S15] J. Li, F. Ye, S. Vaziri, M. Muhammed, M. C. Lemme et al., Efficient inkjet printing of graphene. Adv. Mater. **25**, 3985 (2013). https://doi.org/10.1002/adma.201300361

[S16] Y. Xu, M. G. Schwab. A. J. Strudwick, I, Hennig, X. Feng et al., Screen-printable thin film supercapacitor device utilizing graphene/polyaniline inks. Adv. Energy Mater. **3**, 1035 (2013). https://doi.org/10.1002/aenm.201300184

[S17] K. Arapov, E. Rubingh, R. Abbel, J. Laven, G. de With et al., Conductive screen printing inks by gelation of graphene dispersions. Adv. Funct. Mater. **26**, 586 (2016). https://doi.org/10.1002/adfm.201504030

[S18] L. W. T. Ng, X. Zhu, G. Hu, N. Macadam, D. Um et al., Conformal printing of graphene for single- and multilayered devices onto arbitrarily shaped 3D surfaces. Adv. Funct. Mater. **29**, 1807933 (2019). https://doi.org/10.1002/adfm.201807933

[S19] Y. Sui, A. Hess-Dunning, P. Wei, E. Pentzer, R. M. Sankaran et al., Electrically conductive, reduced graphene oxide structures fabricated by inkjet printing and low temperature plasma reduction. Adv. Mater. Technol. **4**, 1900834 (2019). https://doi.org/10.1002/admt.201900834

[S20] E. B. Secor, T. Z. Gao, A. E. Islam, R. Rao, S. G. Wallace et al., Enhanced conductivity, adhesion, and environmental stability of printed graphene inks with nitrocellulose. Chem. Mater. **29**, 2332 (2017). https://doi.org/10.1021/acs.chemmater.7b00029

[S21] H. Ding, P. He, J. Yang, C. Liu, H. Zho et al., Water-based highly conductive graphene inks for fully printed humidity sensors. J. Phys. D: Appl. Phys. **53**, 455304 (2020). https://doi.org/10.1088/1361-6463/aba78a

[S22] H. Chen, Y. Zhang, Y. Ma, S. Chen, Y. Wu et al., Sand-milling exfoliation of structure controllable graphene for formulation of highly conductive and multifunctional graphene inks. Adv. Mater. Interfaces **8**, 2000888 (2020). https://doi.org/10.1002/admi.202000888

[S23] K. Arapov, E. Rubingh, R. Abbel, J. Laven, G. de With et al., Conductive screen printing inks by gelation of graphene dispersions. Adv. Funct. Mater. **26**, 586 (2016). https://doi.org/10.1002/adfm.201504030

[S24] S. Majee, M. Song, S.L. Zhang, Z.B. Zhang, Scalable inkjet printing of shear-exfoliated graphene transparent conductive films. Carbon **102**, 51 (2016). https://doi.org/10.1016/j.carbon.2016.02.013

[S25] E.B. Secor, P.L. Prabhumirashi, K. Puntambekar, M.L. Geier, M.C. Hersam, Inkjet printing of high conductivity, flexible graphene patterns. J. Phys. Chem. Lett. **4**, 1347 (2013). https://doi.org/10.1021/jz400644c
